# Editorials: voices of a scientific journal

**DOI:** 10.1590/0102-311XEN183225

**Published:** 2025-11-07

**Authors:** Luciana Correia Alves, Luciana Dias de Lima, Marilia Sá Carvalho

**Affiliations:** 1 Instituto de Filosofia e Ciências Humanas, Universidade Estadual de Campinas, Campinas, Brasil.; 2 Escola Nacional de Saúde Pública Sergio Arouca, Fundação Oswaldo Cruz, Rio de Janeiro, Brasil.; 3 Programa de Computação Científica, Fundação Oswaldo Cruz, Rio de Janeiro, Brasil.

In the scientific publication process, it is common for authors to wonder or ask editors about the reasons that led to the rejection of their articles without the peer review step.

Scientific publication has undergone substantial changes that have redefined the role of researchers and periodicals. One of the main effects of these changes is the intensification of the demand for article production and submission, accompanied by an increase in productivity, which, in many cases, favors quantity over quality and can compromise scientific integrity. As a consequence of this phenomenon, a scenario characterized by a true *paper boom* emerges, placing direct pressure on all stakeholders involved in the editorial process. With the exponential growth in the quantity of papers that scientific journals receive on a monthly basis, especially those that do not charge submission or publication fees, it has become necessary to readjust editorial processes.

Changes in established procedures or practices do not necessarily constitute a definitive solution to the challenges of scientific publication but do constitute a necessary strategy to ensure the quality of the good workflow in scientific communication. Thus, authors also share the responsibility of adopting rigorous care so that their papers can be properly assessed and understood ^1^. The submission of an academic article is often a time of excitement but requires patience and meticulous attention to detail in order to minimize the occurrence of errors ^1^.

At CSP, all manuscripts undergo an initial assessment of the suitability of the topic, methodological relevance, and alignment with the objectives and scope of the journal ^2^. As we receive many more submissions than we can send for peer review, it is not possible to forward all papers to this stage. Therefore, we prioritize articles that make innovative, original contributions, offer well-organized information and up-to-date references, and are of broader interest to our readership. It is also essential for all papers to have some form of social relevance.

Rejection at this stage does not imply failures in all aspects of the article, such as quality, methods, or other assessment criteria. We often receive papers on recurring topics that end up repeating discussions already extensively explored and published. In such cases, we give preference to those that break from the “more of the same” approach, offering novel contributions, whether theoretical, methodological, or empirical ^3^. When this does not occur, we understand that the manuscript is not a priority at the moment and may be better received by other periodicals. The swiftness of this decision enables authors to direct their article to another dissemination vehicle in a timely manner. Authors can certainly appeal editorial decisions at any stage of the review. However, at this specific stage, the request for reviews faces limitations, precisely due to the reason that led to the rejection.

Editorials serve an essential function and are a key element of the scientific publication process. They play the role of expressing the reflections of the Editorial Board on selected articles, a specific topic, emerging trends, or editorial policies. Thus, editorials constitute the “voice of the journal” - the space in which its identity is manifested, its values ​​are revealed, the paths it intends to offer readers and authors are delineated, and the perspectives of its editors are recorded ^4^.

Before writing a manuscript, authors should first choose the journal in which they intend to publish. For such, it is essential to perform a careful reading of editorials and determine whether the topic of the paper is aligned with the fields and central interests of the periodical. It is also important to note whether and how often the topic has previously been covered: the fact of having publications indicates pertinence, but, in some cases, can constitute saturation of the topic, thus reducing editorial interest at the time. When this alignment is not achieved, the most appropriate course of action is to submit the paper to another periodical whose editorial line is closer to the communication that the author wishes to establish.

The concept of originality in the publication process is not limited to the novelty of the data or the objectives of the study. It involves, above all, the ability to understand how the phenomenon investigated develops over time and to establish its relationship with the reality of the affected populations. Striking examples of this are studies on the COVID-19 pandemic. At least initially, CSP gave priority to more up-to-date analyses of the impacts of the pandemic over studies that, although situated in unprecedented contexts, addressed dynamics already widely described in other scenarios. Naturally, this is not a binary criterion, nor is it unquestionable, and each submission is assessed on its specificities.

Another important aspect concerns academic-scientific writing itself. Different discursive genres make up the routine of researchers: from the initial project or proposal to the dissemination of the results of a study in theses, articles, and reports. However, due to the pressure for productivity and increasingly short deadlines, many authors end up submitting articles cut directly from other documents - or, even worse, do not have an adequate background to distinguish one genre from another. The rigor CSP maintains regarding this aspect is a form of resistance to the naturalization of this unbridled productivity.

This editorial invites readers to familiarize themselves with - or revisit - the CSP guidelines presented throughout its editorials, both the oldest and most recent. The editorial space serves as a beacon, guiding the submission of studies that are aligned with the journal’s profile and that contribute to the advancement of scientific knowledge, while remaining open to the plurality of paths and perspectives that characterize the field of Collective Health.
