# Supplementary material for: Editorials: voices of a scientific journal
Source: Cad Saude Publica. 2025 Nov 7;41(10):e00183225. doi: 10.1590/0102-311XEN183225 (PMC12600015; doi:10.1590/0102-311XEN183225)
Supplement: Translation

# Translation: Editoriais: vozes de uma revista científica

**DOI:** 10.1590/0102-311XPT183225

**Authors:** Luciana Correia Alves, Luciana Dias de Lima, Marilia Sá Carvalho

**Affiliations:** 1 Instituto de Filosofia e Ciências Humanas, Universidade Estadual de Campinas, Campinas, Brasil.; 2 Escola Nacional de Saúde Pública Sergio Arouca, Fundação Oswaldo Cruz, Rio de Janeiro, Brasil.; 3 Programa de Computação Científica, Fundação Oswaldo Cruz, Rio de Janeiro, Brasil.

No processo de publicação científica, é comum que autores se questionem ou questionem os editores sobre os motivos que levaram à recusa de seus artigos sem a etapa de revisão por pares. 

Historicamente, a publicação científica passou por alterações substanciais que redefiniram o papel dos pesquisadores e dos periódicos. Entre os principais efeitos dessas alterações, destaca-se a intensificação da exigência de produção e submissão de artigos, acompanhada pelo aumento da produtividade que, em muitos casos, favorece a quantidade em detrimento da qualidade e que pode comprometer a integridade científica. Como consequência desse fenômeno, observa-se um cenário caracterizado por um verdadeiro *paper boom*, que gera uma pressão direta sobre todos os atores envolvidos no processo editorial. Com o crescimento exponencial da quantidade de artigos que as revistas científicas recebem mensalmente, especialmente aquelas que não cobram taxas de envio ou publicação, tornou-se necessário readequar os processos editoriais.

Mudanças de procedimentos ou de práticas estabelecidas não representam, necessariamente, uma solução definitiva para os desafios da publicação científica. No entanto, representam uma estratégia necessária para garantir a qualidade do bom fluxo de trabalho na comunicação científica. Nesse contexto, também recai sobre os autores a responsabilidade de adotar cuidados rigorosos, de modo que seus textos possam ser avaliados e compreendidos adequadamente ^1^. A submissão de um artigo acadêmico costuma ser um momento de entusiasmo, mas demanda paciência e atenção minuciosa aos detalhes, de modo a reduzir ao máximo a ocorrência de erros ^1^.

Em CSP, todos os manuscritos passam por uma avaliação inicial quanto à adequação do tema, relevância metodológica e alinhamento com os objetivos e escopo da revista ^2^. Por recebermos muito mais submissões do que podemos enviar para avaliação por pares, não é possível encaminhar todos os trabalhos para essa etapa. Nesse sentido, priorizamos artigos que tragam contribuições inovadoras e originais, informações bem organizadas, referências atualizadas e de interesse mais amplo para nossa comunidade de leitores. É primordial que os artigos tenham algum tipo de relevância social. 

A recusa nessa etapa não significa falhas em todos os aspectos do artigo, tais como qualidade, métodos ou outros critérios de avaliação. Muitas vezes, recebemos trabalhos sobre temas recorrentes, que acabam por repetir discussões já amplamente exploradas e publicadas. Nesses casos, damos preferência àqueles que rompem com o “mais do mesmo”, oferecendo contribuições novas, sejam teóricas, metodológicas ou empíricas ^3^. Quando isso não ocorre, entendemos que o manuscrito não constitui prioridade no momento e pode ter melhor acolhida em outros periódicos. A celeridade na emissão dessa decisão permite que os autores direcionem oportunamente seu artigo a outro veículo de divulgação. Certamente os autores podem recorrer das decisões editoriais, em qualquer etapa da avaliação. Entretanto, nessa etapa específica, a solicitação de pareceres encontra limitações, justamente em função do motivo que fundamenta a recusa. 

Os editoriais desempenham uma função essencial e são elemento chave do processo de publicação científica. Cumprem o papel de expressar as reflexões do Conselho Editorial sobre os artigos selecionados, sobre um tema específico, sobre tendências emergentes ou sobre políticas editoriais. Representam, portanto, “a voz da revista” − o espaço em que se manifesta sua identidade, se revelam seus valores, se delineiam os caminhos que pretende oferecer a leitores e autores, e se registram as perspectivas de seus editores ^4^.

Antes de elaborar um manuscrito, é recomendável que o autor escolha previamente a revista em que pretende publicar. Para isso, é fundamental realizar uma leitura atenta dos editoriais e verificar se o tema do artigo dialoga com as áreas e os interesses centrais da publicação. Também é importante observar se o assunto já foi tratado anteriormente e com que frequência: o fato de haver publicações indica pertinência, mas, em alguns casos, pode sinalizar saturação do tema, reduzindo o interesse editorial no momento. Quando não há esse alinhamento, a alternativa mais adequada é direcionar o texto a outro periódico cuja linha editorial se aproxime mais da comunicação que se deseja estabelecer.

O conceito de originalidade no processo de publicação não se limita ao ineditismo dos dados ou dos objetivos do estudo. Ele envolve, sobretudo, a capacidade de compreender como o fenômeno investigado se desenvolve ao longo do tempo e de estabelecer sua relação com a realidade das populações afetadas. Um exemplo marcante são os estudos sobre a pandemia de COVID-19. Ao menos num primeiro momento, CSP deu prioridade a análises mais atualizadas dos impactos da pandemia em comparação com estudos que, embora situados em contextos inéditos, tratavam de dinâmicas já amplamente descritas em outros cenários. Naturalmente, esse não é um critério binário, tampouco inquestionável, e cada submissão é avaliada em suas especificidades. 

Outro aspecto importante diz respeito à própria escrita acadêmico-científica. Diferentes gêneros discursivos compõem a rotina do pesquisador: do projeto ou proposta inicial até a divulgação dos resultados de uma pesquisa em teses, artigos e relatórios. Muitas vezes, porém, em função da pressão por produtividade e de prazos cada vez mais curtos, muitos autores acabam submetendo artigos recortados diretamente de outros documentos - ou, pior ainda, não chegam a ter formação adequada para distinguir um gênero de outro. O rigor mantido por CSP quanto a esse aspecto é uma forma de resistência à naturalização dessa produtividade desmedida. 

Este editorial convida os leitores a conhecer - ou revisitar - as diretrizes de CSP, apresentadas ao longo de seus editoriais, tanto as mais antigas quanto as mais recentes. O espaço editorial funciona como um farol, orientando a submissão de estudos alinhados ao perfil da revista e que contribuam para o avanço do conhecimento científico, mantendo-se, ao mesmo tempo, aberto à pluralidade de percursos e perspectivas que caracterizam o campo da Saúde Coletiva.

# Translation: Editoriales: voces de una revista científica

**DOI:** 10.1590/0102-311XES183225

**Authors:** Luciana Correia Alves, Luciana Dias de Lima, Marilia Sá Carvalho

**Affiliations:** 1 Instituto de Filosofia e Ciências Humanas, Universidade Estadual de Campinas, Campinas, Brasil.; 2 Escola Nacional de Saúde Pública Sergio Arouca, Fundação Oswaldo Cruz, Rio de Janeiro, Brasil.; 3 Programa de Computação Científica, Fundação Oswaldo Cruz, Rio de Janeiro, Brasil.

En el proceso de publicación científica es común que los autores se cuestionen a sí mismos o a los editores sobre las razones que llevaron al rechazo de sus artículos sin la etapa de revisión por pares.

Históricamente, la publicación científica ha sufrido cambios sustanciales que han redefinido el papel de los investigadores y de las revistas. Entre sus principales efectos destacan la intensificación del requisito de producir y enviar artículos, seguido de un aumento de la productividad, que favorece, en muchos casos, la cantidad sobre la calidad y puede comprometer la integridad científica. Como consecuencia de este fenómeno, se presenta un escenario caracterizado por un verdadero *paper boom*, que genera una presión directa sobre todos los actores involucrados en el proceso editorial. Dado el crecimiento en el número de artículos que las revistas científicas reciben mensualmente, especialmente aquellas que no cobran tarifas de envío o publicación, es necesario reajustar los procesos editoriales.

Los cambios en los procedimientos o en las prácticas que se han establecido no representan necesariamente una solución definitiva a los desafíos de la publicación científica. Aunque sí representan una estrategia necesaria para asegurar la calidad del buen flujo de trabajo en la comunicación científica. En este contexto, los autores también son responsables de adoptar un cuidado riguroso para que sus textos sean claros y evaluados adecuadamente ^1^. El envío de un artículo académico suele ser un momento de entusiasmo, que requiere paciencia y atención meticulosa a los detalles, con el fin de reducir al máximo los errores ^1^.

En CSP, todos los manuscritos se someten a una evaluación inicial sobre la adecuación del tema, la relevancia metodológica y la alineación con los objetivos y el alcance de la revista ^2^. Debido a la gran cantidad de los manuscritos recibidos más de lo que podemos enviar a la revisión por pares, no es posible pasar todos los trabajos a esta etapa. Por ello, priorizamos artículos que tengan aportes innovadores y originales, información bien organizada, referencias actualizadas y de mayor interés para nuestros lectores. Es crucial que los artículos tengan algún tipo de relevancia social.

El rechazo en esta etapa no significa fallas en todos los aspectos del artículo, como en la calidad, en los métodos u otros criterios de evaluación. Muchas veces recibimos artículos sobre temas recurrentes, pero terminan repitiendo discusiones que ya han sido ampliamente exploradas y publicadas. En estos casos, elegimos a aquellos que rompen con “más de lo mismo” al contribuir con nuevas aportaciones, ya sean teóricas, metodológicas o empíricas ^3^. Cuando esto no ocurre, entendemos que el manuscrito no es una prioridad en aquel momento y puede ser mejor aceptado en otras revistas. La rapidez en la emisión de esta decisión permite que los autores envíen oportunamente su artículo a otro medio de divulgación. Ciertamente, los autores pueden apelar las decisiones editoriales en cualquier etapa de la evaluación. Sin embargo, en esta etapa específica, la solicitud de opiniones es limitada, precisamente debido al motivo del rechazo.

Los editoriales juegan un papel clave y son un elemento importante del proceso de publicación científica. Desempeñan el papel de expresar las reflexiones del Consejo Editorial sobre los artículos seleccionados, sobre un tema específico, sobre las tendencias emergentes o sobre las políticas editoriales. Por lo tanto, representan “la voz de la revista”: el espacio en que manifiesta su identidad, revela sus valores, describe los caminos que pretende ofrecer a los lectores y autores, y registra las perspectivas de sus editores ^4^.

Antes de preparar un manuscrito, se recomienda que el autor elija previamente la revista en la que pretende publicar. Para ello, es fundamental leer atentamente los editoriales y verificar si la temática del artículo dialoga con las áreas centrales e intereses de la publicación. También es importante tener en cuenta si el tema se ha tratado anteriormente y con qué frecuencia: el hecho de que haya publicaciones indica pertinencia, pero, en algunos casos, puede evidenciar la saturación del tema, lo cual reduce el interés editorial en este momento. Cuando no existe esta alineación, la alternativa más adecuada es enviar el texto a otra revista cuya línea editorial esté más en consonancia con la comunicación que se pretende establecer.

El concepto de originalidad en el proceso de publicación no se limita a los datos o los objetivos del estudio. Implica, sobre todo, la capacidad de entender cómo se desarrolla a lo largo del tiempo el fenómeno investigado y de establecer su relación con la realidad de las poblaciones afectadas. Un ejemplo son los estudios sobre la pandemia de la COVID-19. Al menos al principio, CSP dio prioridad a análisis más actualizados de los impactos de la pandemia en comparación con estudios que, aunque situados en contextos sin precedentes, trataban dinámicas ya ampliamente descritas en otros escenarios. Por supuesto que este no es un criterio binario ni incuestionable, y cada presentación de los manuscritos se evalúa en sus especificidades.

Otro aspecto importante se refiere a la escritura académico-científica. Diferentes géneros discursivos conforman la rutina del investigador: desde el proyecto o propuesta inicial hasta la difusión de los resultados de una investigación en tesis, artículos e informes. Sin embargo, muchas veces, debido a la presión por la productividad y los plazos cada vez más cortos, muchos autores terminan presentando artículos cortados directamente de otros documentos o, lo que es peor, no tienen la formación adecuada para distinguir un género de otro. El rigor que mantiene CSP al respecto es una forma de resistencia a la naturalización de esta excesiva productividad.

Este editorial les invita a sus lectores a conocer -o revisar- las directrices de CSP presentadas a lo largo de sus editoriales, tanto las más antiguas como las más recientes. El espacio editorial actúa como faro que orienta el envío de estudios alineados con el perfil de la revista y que contribuyan al avance del conocimiento científico, al mismo tiempo que permanece abierto a la pluralidad de caminos y perspectivas que caracterizan el campo de la Salud Pública.
